# Obtaining Cocrystals by Reaction Crystallization Method: Pharmaceutical Applications

**DOI:** 10.3390/pharmaceutics13060898

**Published:** 2021-06-17

**Authors:** Isabela Fanelli Barreto Biscaia, Samantha Nascimento Gomes, Larissa Sakis Bernardi, Paulo Renato Oliveira

**Affiliations:** Post-Graduation Program in Pharmaceutical Sciences, Department of Pharmacy, Universidade Estadual do Centro-Oeste/UNICENTRO, Guarapuava 85040-080, PR, Brazil; isabelafbarreto@hotmail.com (I.F.B.B.); samanthagomes@gmail.com (S.N.G.); larissa.sb@gmail.com (L.S.B.)

**Keywords:** pharmaceutical cocrystal, cocrystal, reaction crystallization method, cocrystallization, solubility

## Abstract

Cocrystals have gained attention in the pharmaceutical industry due to their ability to improve solubility, stability, in vitro dissolution rate, and bioavailability of poorly soluble drugs. Conceptually, cocrystals are multicomponent solids that contain two or more neutral molecules in stoichiometric amounts within the same crystal lattice. There are several techniques for obtaining cocrystals described in the literature; however, the focus of this article is the Reaction Crystallization Method (RCM). This method is based on the generation of a supersaturated solution with respect to the cocrystal, while this same solution is saturated or unsaturated with respect to the components of the cocrystal individually. The advantages of the RCM compared with other cocrystallization techniques include the ability to form cocrystals without crystallization of individual components, applicability to the development of in situ techniques for the screening of high quality cocrystals, possibility of large-scale production, and lower cost in both time and materials. An increasing number of scientific studies have demonstrated the use of RCM to synthesize cocrystals, mainly for drugs belonging to class II of the Biopharmaceutics Classification System. The promising results obtained by RCM have demonstrated the applicability of the method for obtaining pharmaceutical cocrystals that improve the biopharmaceutical characteristics of drugs.

## 1. Introduction

The enhancement of essential properties of an active pharmaceutical ingredient (API)—for example, bioavailability, solubility, flow properties, and thermal stability—are essential to new technological developments in the pharmaceutical industry [1]. Drug molecules with limited aqueous solubility have a slow dissolution in biological fluids as well as insufficient systemic exposure and suboptimal efficacy in patients, and for this reason, they have been the subject of numerous research studies by innovative companies [2]. The development of new pharmaceutical formulations aims to improve ingredient handling during processing, improve storage stability, and increase the dissolution of APIs [3].

Pharmaceutical solids can be generally classified into amorphous or crystalline forms. The component atoms, ions, or molecules in amorphous solids have no ordering, while in crystalline solids they are arranged in a regular order, repeating the pattern in three dimensions [4]. The structures in which a drug may exist are fundamental for defining its physical, chemical, mechanical, and biopharmaceutical properties [5]. Cocrystal crystal lattice is formed through non-ionic [3] and non-covalent interactions between the API and the coformer that allow changes in the physical properties of the drug without altering its mechanism of action [6].

Over the last years, the perception of cocrystal pharmaceutical engineering has gained great visibility [7]. A variety of cocrystal formation techniques are used in the pharmaceutical field, such as slow evaporation [8], solution crystallization [9], the anti-solvent method [10], mechanochemistry [11], melt crystallization [12], Sonic Slurry™ [13], solvo-thermal synthesis [14], the hot-melt extrusion method [15], the spray drying technique [16], supercritical solvent cocrystallization [17], and the reaction crystallization method (RCM) [18].

This review highlights the mechanism by which the reaction crystallization method forms cocrystals and demonstrates how the components of the reaction can guide the formation, formulation, and development of cocrystals. The main objective is to demonstrate the various successful studies and to emphasize the advantage of this specific cocrystalization method for the pharmaceutical industry.

## 2. Pharmaceutical Cocrystals

Cocrystals are multicomponent solids that contain two or more neutral molecules, solid at room temperature [19], in stoichiometric ratio within the same crystal lattice [20]. They are composed of an API and a cocrystallizing agent (coformer), typically a small organic compound [21], generally regarded as safe, which may be another drug or a non-toxic molecule [20]. Cocrystals of poorly aqueous soluble APIs and highly soluble coformers have been shown to impart a solubility advantage that is orders of magnitude higher than the parent drug [22], as well as a dissolution advantage and increased bioavailability [23].

Multicomponent solid forms include both crystalline and amorphous systems. Crystallinity gives cocrystals a stability advantage compared with amorphous materials. In comparison with salts that rely on ionic interactions [24], cocrystals are formed by reversible and non-covalent interactions [25] between drug and coformer functional groups [7]. Different from solvates, cocrystal components are solid at room temperature.

Because cocrystals have great potential for the pharmaceutical industry and because a few are now on the market [26], the US Research and Evaluation Center of the Food and Drug Administration (FDA) has issued a guidance for industry about the regulatory classification of pharmaceutical cocrystals [27]. In this document, the FDA defines cocrystals as crystalline solids composed of two or more molecules in the same crystal lattice, being a molecular complex dissociable from API-excipient, where the coformer is the excipient. In order to characterize the cocrystal, the FDA requires evidence that both the API and the coformer exist in their neutral states and that no ionic interaction is used to associate the two substances [27].

The EMA (European Medicines Agency) has also published a discussion paper about the use of cocrystals in medicinal products, which states that cocrystals are a versatile tool that can be used to obtain more appropriate solid-state properties. The document defines cocrystals as homogeneous crystalline structures constituted by two or more components in a definitive stoichiometric relation where the arrangement in the crystalline lattice is not based on ionic bonds and requires that the formation of the cocrystal is demonstrated by means of suitable analytical techniques [28].

Cocrystal formation is characterized as the crystallization of a single phase in a stoichiometric ratio according to the following equilibrium reaction for a binary cocrystal *A_y_B_z_* [18,29]:

(1) $${\left(A_{y}B_{z}\right)}_{solid}\;\underset{⇋}{K_{\mathrm{sp}}}\;yA_{solution}+zB_{solution}$$

where *A* and *B* represent drug and coformer, and *z* and *y* represent their respective stoichiometric coefficients. The reaction describes cocrystal precipitation (right to left) and dissolution (left to right).

The solubility product (*K*_sp_) is the thermodynamic equilibrium constant of the reaction and is given by the product of activity coefficients (*γ*) of components *A* and *B* multiplied by the component concentrations [29]:

*K*_sp,a_*= γ_A_*[*A*]^y^ *γ_B_*[*B*]^z^ ≈ *K*_sp_ = [*A*]^y^ [*B*]^z^ (2)

where [*A*] and [*B*] are the molar concentrations of each cocrystal component at equilibrium. Under ideal conditions *γ* is equal to 1, and the activity-based solubility product is replaced by the concentration product *K*_sp_.

## 3. Techniques for Obtaining Cocrystals

Several different strategies have been reported by researchers for preparing cocrystals [30]. One classic technique for obtaining cocrystals is crystallization in solution. This traditional method of crystallization from solution is carried out with a suitable solvent in which all the components are soluble, which promotes the formation of the cocrystals. Stoichiometric amounts of the components are dissolved in a solvent, which can be heated to facilitate dissolution and then evaporated [31]. One of the disadvantages of the method is the large number of possible solvents that are necessary, which requires several screening experiments [32]. Another approach is the solvo-thermal technique, where the supersaturation of the cocrystal occurs through temperature changes [14,33]. For this method to be successful, a solvent screening is necessary, in which the substances have similar solubilities in order to minimize individual crystallization [29]. The anti-solvent method can also be used, in which a solvent to which the compound is less soluble is added to another solution, generating supersaturation and favoring the precipitation of solids [10]. The above-mentioned methodologies, however, have a chance of crystallizing the single component phases, thereby reducing the possibility of accessing the multicomponent crystalline phase [18].

In the mechanochemistry methodology, solid-state crystallization occurs through the mechanical activation by grinding. This method can reduce screening [34] and generates cocrystals that are not possible by means of solution [35]. The efficiency of the cocrystallization by grinding can be improved by adding catalytic amounts of solvents [36], which leads to the formation of the cocrystal by altering the molecular mobility of the solid phase and/or interactions in solution [37] and obtains a greater quantitative yield with a higher degree of crystallinity than simple grinding [38].

Cocrystal formation by melting, also known as the technique of Kofler, was used by the inventor to investigate cocrystals in the 1940s [39]. The method consists of the simultaneous fusion of two compounds in a blade, followed by solidification, generating a molecular complex with both components, which under appropriate conditions forms the cocrystal [12]. Some organic liquids with high boiling points such as methyl salicylate or methyl benzoate may be used to create highly concentrated solutions and reduce the melting point of the API used [23].

It is also possible to synthesize cocrystals through Sonic Slurry™ [13], a high-throughput technique performed in well plates [40]. A slurry is a dispersion of solid particles in a liquid phase that comprises one or more solvents in which the chemical substance is not completely soluble. This process is based on generating supersaturation with respect to the cocrystal by subjecting a slurry of reactants to ultrasound pulses for accelerating the process [41,42].

There is also a method based on heat that does not use solvent: the hot-melt extrusion method, in which the cocrystal is prepared by heating the drug and coformers with intense mixing. The heat used allows only the matrix to be melted, and the method also uses a catalytic agent to improve cocrystal formation. The disadvantage of this methodology is that it requires compatibility of the components with the liquefied type and cannot be used for thermally unstable substances [15]. Heating is also used in the spray drying technique, which is a method that transforms a liquid into a dry particulate form through a gas and high temperatures. Cocrystal formation occurs during spray drying in the presence of a carrier excipient. The process is advantageous because it is continuous, controllable, and fast [16].

The supercritical solvent cocrystallization technique uses the solvent power of supercritical CO_2_ to suspend the API and coformer as a slurry in supercritical CO_2_. The solution is quickly depressurized; consequently, the solvent power decreases and produces supersaturation, which leads to crystallization. The process’s disadvantages are limited solubility in supercritical CO_2_ and low yields [17].

As a consequence of the empirical basis of the techniques used in search of cocrystals, a large number of experimental conditions are often tested [5] and transferability to larger scale crystallization processes is restricted [18].

### 3.1. Reaction Crystallization Method

#### 3.1.1. Mechanism

The RCM was described for the first time by Rodríguez-Hornedo et al. The RCM is based on the reduction of the solubility of the molecular complex that forms the cocrystal, generating conditions for its nucleation and crystallization to occur. The driving force for cocrystallization is supersaturation, resulting from the excess addition of the individual components (drug and coformer) in solution, resulting in non-stoichiometric concentrations [18].

**Figure 1:**
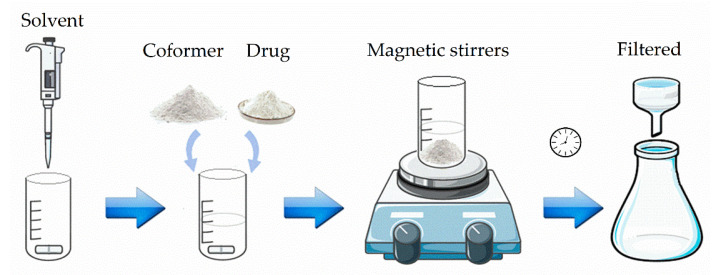
Illustration of cocrystal production by the RCM. First, the solvent is added, then the coformer and the drug. The reaction is kept under magnetic stirring for approximately 48 h and then filtered.

As shown in Figure 1, cocrystal preparation uses saturated solutions of coformer to which an amount of drug above its solubility is added. These solutions can be obtained by dissolving the drug and the coformer in a pure solvent, or in a solvent containing solid components, or by mixing two solutions containing the drug and the coformer already dissolved [31]. As the drug is added into the saturated coformer solution, it dissolves up to its solubility, and cocrystal precipitates out of the solution. The solution or slurry is kept under stirring for the time necessary for the reaction to take place and is then filtered [18,43].

**Figure 2:**
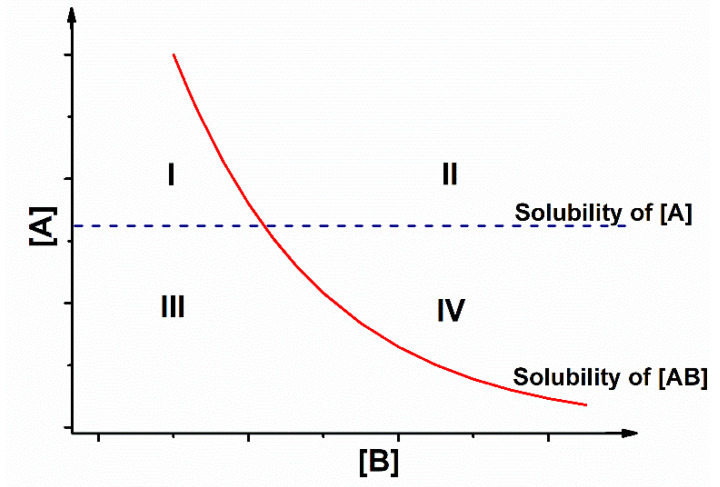
Solubility of an AB cocrystal in solution. [A]: Concentration of the drug; [B]: concentration of the coformer. Lines represent solubilities of drug A and cocrystal AB. Region I: supersaturated solution in relation to the drug and unsaturated in relation to the cocrystal. Region II: supersaturated solution in relation to drug and cocrystal. Region III: unsaturated solution. Region IV: supersaturated solution in relation to the cocrystal and unsaturated in relation to the drug. Source: Adapted from Kuminek et al. (2016) with permission from the publisher.

The differences in the behavior of the drug and the coformer are explained by the composition of the solution, according to the phase diagrams. The phase solubility diagram (Figure 2) shows graphically how cocrystal supersaturation can be generated while component solution concentrations are at or below saturation [44].

In this way, at a certain point the cocrystal and drug solubility curves intersect. Therefore, there is a concentration of coformer (B), in which the solubility of cocrystal (AB) is equal to the solubility of drug (A) and above which the solubility of cocrystal (AB) is less than that of drug (A). This concentration is known as a transition concentration that can be predicted by [43]

*[B]_t_ = S + C_t_ = S + ((K_*sp*_ − S_A_*^2^*)/S_A_)* (3)

where [*B*] is the total coformer concentration at the transition point, *S* is the cocrystal solubility in stoichiometric conditions, *C_t_* is the excess concentration of coformer, and *S_A_* is the drug solubility. This equation predicts that the total concentration of the coformer at the transition point increases as the solubility of the drug decreases or as the *K_*sp*_* increases [43].

The phase diagram has four domains. In order for cocrystallization to occur, the solution must be in region IV, where it is supersaturated with respect to the cocrystal [43,45]. In this way, the drug and the coformer may form the cocrystal through a solution-mediated reaction, which consumes the components of the solution until the process reaches the equilibrium state defined by the cocrystal [18,43].

This graph indicates the regions of thermodynamic stability and which components can dissolve or crystallize. For the development of cocrystals, it is useful to identify the conditions where the phase transformations between drug and cocrystal occur and to control crystallization.

For crystallization to occur, it is necessary to consider the drug and coformer concentrations, which dictate the supersaturation of the cocrystal. There will be two eutectic points (one between cocrystal and drug, and other between coformer and cocrystal) where the solvent content is at its lowest value, which means that the solubility is at its highest value. The cocrystal will only be stable, less soluble than the drug or coformer, at concentrations between the eutectic points. Knowledge of this concentration range, called the cocrystal operating range, is key to designing a successful cocrystallization solution [46].

#### 3.1.2. Influence of the Quantities of Individual Components

In the RCM, cocrystallization is mediated by a solution in which the dissolution of pure drug generates supersaturated conditions with respect to the cocrystal, which leads to the formation of the cocrystal. When one of the reagents is in the solid state and in an amount higher than its solubility value, it is consumed by the cocrystallization reaction, and the final product is the cocrystalline phase [18].

Cocrystallization rates can be assessed by in situ determination of the Raman peak, which changes when the drug changes to cocrystal. The time taken to convert the drug to cocrystal depends on the amounts of the components in the solution. The drug must be added at solubility, and the increase in the concentration of the coformer results in a faster conversion of pure drug to cocrystal as it increases the initial supersaturation in relation to the cocrystal and increases the cocrystallization rate, decreasing the time for transformation into cocrystal [18,47].

Equation (3) shows that the rate of supersaturation and crystallization can be in-creased by increasing the concentration of individual components [48]. The driving force for nucleation and growth is the supersaturation, σ, which is derived from the difference in chemical potential between the supersaturated solution state and the saturated solution state and is expressed by [29]

*σ = ([A][B]/K_*sp*_)*^1*/v*^ (4)

where [*A*] is the concentration of the drug in solution, [*B*] is the concentration of the coformer in solution, v is the number of components of the cocrystal (in a binary cocrystal, 2), and *K_*sp*_* is the product of solubility.

Supersaturation with respect to the cocrystal is dependent on solution composition [48]. It is important to note that it is the dissolved components that determine supersaturation, not the solid phases of the components.

#### 3.1.3. Solvent

The RCM can be used with many solvents, but water and alcohols are the most relevant [29]. Prior knowledge of the cocrystal solubility in pure solvent is useful for predicting the solubility phase diagram as a function of the concentration of the coformer and for determining the conditions under which the crystals dissolve or precipitate [43].

**Figure 3:**
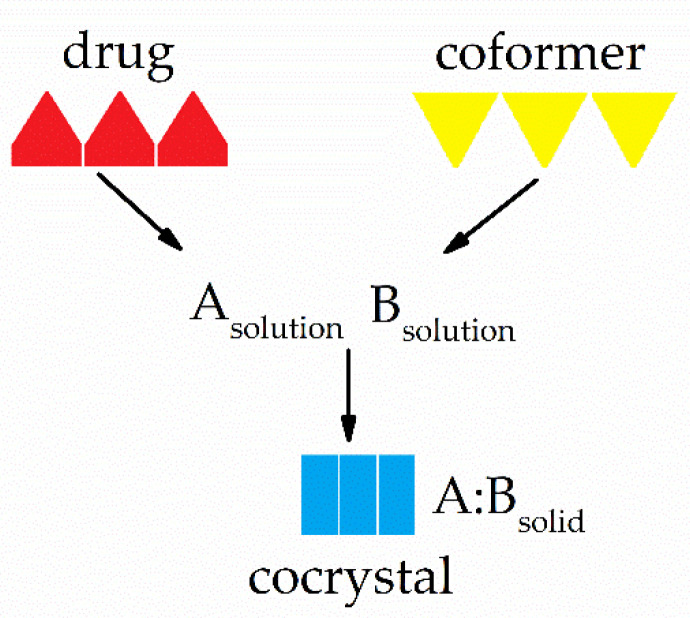
Formation of a cocrystal A:B from the generation of supersaturation by dissolving components A (drug) and B (coformer) in a microphase of solvent. Adapted from Rodriguez-Hornedo et al. (2006).

Cocrystals can be prepared in macrophases by suspending the drug in saturated solutions of coformer at room temperature. The method can also be developed using solvent microphases, that is, only drops of organic solvent, such as ethanol or 2-propanol. In this situation, the cocrystallization reaction takes place via a similar route to suspension in larger amounts of solvent. The drops of solvent added to the components must allow the dissolution of both so that the non-stoichiometric quantities generate the supersaturation necessary for the formation of the cocrystal, as shown in Figure 3 [18].

Like other solution-based methods, the RCM has the disadvantage of needing to separate the formed cocrystals from the solvent [49]. It can also generate undesirable solvated or hydrated cocrystals, in addition to generating waste if non-environmentally friendly solvents are used.

#### 3.1.4. Advantages

The RCM offers significant improvements over other methods of cocrystal production. Since the RCM is based on thermodynamically stable crystal conditions, it is an attractive process for any scale of reaction [50].

In a study that investigated crystallization methods capable of producing cocrystals on an industrial scale, the RCM was reported to produce 250 g/day of cocrystals [51]. The RCM is a good approach for commercial scale due to the availability of solution crystallization equipment (large stirred-tank reactors) in pharmaceutical factories [52]. Furthermore, by changing the process parameters (solvents, addition of solubilizing agents and polymers), different pathways can be achieved in the phase solubility diagram, which requires strict control but allows the origination of different cocrystals [53]. Other techniques do not present advantages for scaling up and can compromise the purity of the formed cocrystals, such as mechanochemistry, which can generate amorphization and crystal defects due to intensive energy input [54]. Techniques that involve elevated temperature during crystallization can also limit their suitability for thermally unstable compounds [4].

For successful use in the pharmaceutical formation of tablets, it is necessary that the cocrystals be stabilized by the components of the formulation. Ullah et al. (2015) obtained cocrystals of carbamazepine and succinic acid by the RCM and developed formulations using three different polymers in the formulation. The results showed a 16% improvement in the in vitro dissolution rate in the simulated intestinal fluid, and the bioavailability was significantly better than the commercial-based tablet [55]. In another study, it was concluded that an adequate formulation was necessary to take advantage of the increased solubility of the 1:1 danazol:vanillin cocrystal obtained by RCM. The researchers observed that the aqueous suspension of the cocrystal showed an in vivo improvement of 1.7 times greater in the area under the curve, while the formulated aqueous suspension containing 1% vitamin E-TPGS and 2% hydroxypropylcellulose improved the drug’s bioavailability by more than 10 times [56].

The RCM also has the advantage of forming cocrystals without crystallizing individual components. Another point is that this technique can be performed on a variety of solvents, which makes it more environmentally friendly since there is no need to select solvents that correspond to the solubilities of the reagents for this approach to be successful [18].

In addition, the RCM is applicable for developing rational techniques in situ for the screening of high-quality cocrystals. In situ monitoring of cocrystal formation by the RCM might be used to effectively control and scale-up cocrystallization processes. This technique was demonstrated by Gagniere et al., where the process monitoring was carried out using attenuated total reflectance Fourier-transform infrared (ATR-FTIR) spectroscopy [57]. The study presents estimates of the solute concentrations of both carbamazepine (CBZ) and nicotinamide (NCT) at different temperatures, and with the benefit of a spectroscopy, solute concentrations could be monitored during the process.

The RCM may also be used for cocrystal screening in microliter volumes by in situ Raman microscopy. Jayasankar et al. reported cocrystallization results at constant relative humidity in binary and ternary mixtures of cocrystal components with a deliquescent additive that was not consumed by the reaction. The influence of the additive, the relative humidity, and the composition of the solid mixture on the rate of cocrystal formation was studied by on-line Raman spectroscopy during deliquescence [58]. Solid phase changes can be monitored in situ by Raman microscopy or other appropriate method [29].

#### 3.1.5. Pharmaceutical Cocrystals Reported in the Literature Obtained by the RCM

Several studies using the RCM from 2006 to date are available in the scientific literature, and some of them are summarized in Table 1. As it can be seen, the great majority of the drugs used to produce cocrystals belong to class II of the Biopharmaceutical Classification System (BCS). The BCS is based on drug aqueous solubility and intestinal permeation. Drug molecules belonging to class II are those that present low solubility and high permeability [59,60]. Despite the more than 30% of marketed drugs that fall into class II of the BCS, these drug molecules are frequently withdrawn from drug development because of their poor aqueous solubility [61]. Therefore, it is important to develop new pharmaceutical forms that increase the solubility of these drugs.

However, the cocrystals obtained by the RCM also have advantages for drugs from the other classes, as can be seen in Table 1 and as discussed in the following sections.

##### Class II

Rodríguez-Hornedo et al. predicted cocrystal solubility using cocrystal *K*_sp_ Equation (2) and found an excellent agreement with the experimentally measured solubility [14]. In this same study, the results suggest a solution-mediated transformation where dissolution of pure drug creates supersaturated conditions with respect to cocrystal and leads to cocrystallization. This study also indicates that a wide range of solvents can be used for cocrystallization and that solvents need not be limited by the different solubilities of the components, as exemplified by the studied solvents ethanol, 2-propanol, and ethyl acetate.

Childs et al. developed a cocrystal screening where several solvents were presaturated with the coformers of interest, after which the solid drug (in amounts above its solubility) was added into those solutions. Experiments using the RCM generated CBZ cocrystals in water with all carboxylic acids coformers used, except adipic acid [40]. The success of cocrystal formation having water as solvent was a significant and unpredicted finding since this is a solvent well known to form carbamazepine dihydrate. The stability of the cocrystal in water varied depending on the coformer used, suggesting that the aqueous solubility of the coformer can be an important indicator of the stability and solubility of the cocrystal. The study also shows the importance of considering solution concentrations of coformer when selecting solvents for screening processes and crystallization.

Another parameter that must be considered regarding the solubility of the cocrystal is the pH of the solution. In solutions where the cocrystal components ionize, its solubility will be modulated by the extent of ionization [29]. Alhalaweh et al. studied the pH-dependent solubility of indomethacin–saccharin (IND/SAC) and CBZ/saccharin (CBZ/SAC) cocrystals. These cocrystals increased the solubility and conferred a different pH solubility-dependence different than the drugs. The IND/SAC cocrystal showed an increase in solubility of 13–65 times greater than the pure drug, and at pH values from 1 to 3 the CBZ/SAC cocrystal was 2–10 times more soluble than CBZ-hydrated. These results demonstrate leveraging by solvation and not by crystal lattice effects [61]. A further experiment also concluded that the solubility and stability of the gabapen-tin-3-hydroxybenzoic acid cocrystal are pH dependent [68].

The solubility of CBZ cocrystals with several coformers (theophylline, caffeine, nicotinamide, malonic acid, glutaric acid, saccharin, oxalic acid, succinic acid, salicylic acid) were predicted from measurement of eutectic concentrations in water, ethanol, isopropanol, and ethyl acetate. The results showed an increase of 2–152 times in cocrystal solubility compared with the stable CBZ dihydrate form. This same research showed that the solubility of the cocrystal is modulated by the solubility of its individual components, that the eutectic concentration of the coformer increases with the solubility of the cocrystal, and that the use of a coformer 10 times more soluble than the drug results in a cocrystal with a solubility advantage [45].

One study explained through mathematical models how cocrystal solubility is influenced by pH, pKa, and coformer concentration [63]. It was demonstrated that a non-ionizable drug (CBZ) generated cocrystals with pH-dependent solubility, with the use of ionizable coformers (salicylic acid and 4-aminobenzoic acid). The research also found that the use of acidic coformers increased solubility with increasing pH, while the use of amphoteric coformers decreased solubility as the pH approached the range between their pKa values. It also demonstrated that the greater the solubility of the cocrystal, the higher the concentrations of coformer that are needed to maintain the stability of the cocrystal.

An interesting cocrystal obtained by the RCM was 6-Mercaptopurine/isonicotinamide [74]. This drug is used as an antitumor agent, mainly in cases of acute lymphoblastic leukemia. The results showed a solubility of the cocrystal from 1.7 to 2.3 times greater than the pure drug in three different buffers, and the in vitro and in vivo studies showed that the cocrystal has a better dissolution rate and bioavailability in an animal model. The maximum concentration and time were 118.1 ng/mL and 1.6 h for the drug and 130.9 ng/mL and 1.2 h for the cocrystal, resulting in an increase in bioavailability of 168.7%.

The solubilization of seven different cocrystals obtained by the RCM was evaluated in biorelevant media in a study by Lipert et al. [65]. The results of the solubility of the cocrystal in acetate buffer at pH 5.00 and simulated intestinal fluid in fed state (FeSSIF) showed improvements in both tested media. On the other hand, the amount of solubilized cocrystal was not proportional to the amount of drug solubilized in these media. This is due to the additives present in FeSSIF, which extended the solubility of the components, highlighting the risk of anticipating cocrystal behavior in biorelevant media based on studies carried out in water.

Meloxicam cocrystals with acidic coformers (salicylic acid and maleic acid) were also obtained by RCM, and solubility and thermodynamic stability were determined [73]. The results indicate that meloxicam–salicylic acid cocrystal showed an increase of 18 to 146 times in meloxicam solubility in a pH range of 1 to 8, whereas the cocrystal meloxicam–maleic acid increased the solubility from 305 to 41,689 times in the same range of pH. The stability study was carried out using additives such as polymers and solubilizing agents in the solution that avoided the conversion to the more stable crystalline form of the drug. It was found that the thermodynamic stability of the cocrystal with respect to the drug was dependent on the micellar concentration of the sodium lauryl sulfate additive.

A recent study of lamotrigine (LTG) cocrystals and salts synthesized by the RCM focused on challenging the popular notion that pharmaceutical salts are more soluble than cocrystals [72]. Among the cocrystals and salts evaluated, the LTG/NCT cocrystal were observed to have the greatest aqueous solubility, followed by LTG/hydrochloric acid salt, LTG/saccharin salt, and the less soluble cocrystals LTG/methylparaben and LTG/phenobarbital. It was well explained that the solubility advantage depends on the interplay between the chemistry of both solid and solution phases.

One study attempted to explain the phase behavior of anhydrous/hydrated cocrystals when the coformer modulates both water activity and cocrystal solubility [82]. Stability dependence on solution composition and water activity was studied for theophylline/citric acid anhydrous and hydrated cocrystals. It was demonstrated that phase stability is determined by the activities of water and coformer, and that excipients alter the activity of water, which may affect the anhydro/hydrate eutectic points and phase stability. It was also detected that very water-soluble coformer cocrystals are predisposed to conversion due to moisture adsorption and deliquescence of the coformer, which alters the stability and hygroscopic behavior.

Cocrystallization by the RCM has also been shown to be advantageous in simplifying the control of the crystalline form of an anticancer drug candidate for oral administration, TAK-44 [81]. The drug had been previously obtained only in the form of solvates in solution, which is avoided for pharmaceutical development due to the toxicity of the solvent and its physical instability. TAK-44 cocrystals were developed with L-malic acid and L-tartaric acid. Cocrystals were 2–3 times more soluble in aqueous solutions, stable under stress conditions, and successfully suppressed solvatomorphism.

##### Class III

The RCM also showed effective cocrystals in improving the bioavailability of drugs of class III of the BCS system, which have high solubility and low permeability. In a study with the drug acyclovir, two crystals were obtained with fumaric acid and glutaric acid. The results of the powder dissolution experiments in vitro revealed increased solubility with cocrystal formation. The skin permeability was evaluated in vitro and was shown to increase up to 3 times [62]. The higher permeability can be attributed to the log P values of coformers greater than those of acyclovir and to the reduced drug melting point, which can increase their permeability [83].

In this same study, the increase in permeability was not achieved by the formation of acyclovir salt with maleic acid. In comparison with another study that used slow solvent evaporation and assisted grinding to screen acyclovir cocrystals, several solvents (water, methanol, ethanol, acetonitrile, chloroform, n-hexane, cyclohexane, N-dimethylformamide, and acetic acid) and coformers in molar ratio (dicarboxylic acids, higher fatty acids, amino acids, urea, nicotinamide, and saccharin) were tested; however, just a cocrystal of acyclovir–tartaric acid was reproducibly obtained, and only an increase in solubility was possible [84].

##### Class I

Cocrystal obtained by the RCM can also be a useful approach for BCS class I drugs because despite being highly soluble and permeable, there are other properties that can be improved. A study that evaluated the relationship of the mechanical properties of the compression of paracetamol to tablet formation showed an improved performance in the formation of tablets with paracetamol cocrystals with oxalic acid and 4,4-bipyridine, due to the presence of sliding planes, flat layers, and hydrogen bonds [77].

Cocrystals have also been shown to be efficient for the separation of chiral compounds of ofloxacin in their enantiomers. Two tartaric acid derivatives, O, O′-dibenzoyl- (2S,3S)-tartaric acid (D-DBTA) and O, O′-dibenzoyl- (2R,3R)-tartaric acid (L-DBTA) were used as coformers for the formation of diastereomeric cocrystal pairs in the aqueous phase. The results indicated that D-DBTA selectively cocrystallized with the R-enantiomer (R-OFLX), while L-DBTA selectively cocrystallized with S-enantiomer (S-OFLX) in the aqueous phase. The proposed separation process was environmentally benign, with no addition of organic solvent and efficient for separation of racemic compounds that cannot form salts [76].

Polymorphism is interesting for the pharmaceutical industry because differences in the crystal packing can change a drug’s chemical and physical properties, including solubility and bioavailability. Cocrystals are formed by hydrogen bonds, and a change in these bond motifs can lead to synthon polymorphism [85]. Two polymorphs of the salicylamide cocrystal with oxalic acid have been obtained. Form I of the cocrystal was prepared by three alternative methods—solvent-drop grinding, slurry sonication technique, and RCM. The formation of form II was achieved only by the RCM due to a change in the order of solubilization of the components, which shows the benefits and versatility of this method [79].

## 4. Conclusions

This article focused on the reaction crystallization method for obtaining cocrystals. The RCM is complex to understand, but it is easy, practical, and economical to implement. It can be performed in several laboratories and does not require expensive and specific equipment. In addition, the RCM has several advantages over other cocrystal synthesis techniques. In recent years, several studies have demonstrated the successful use of the RCM to obtain cocrystals, and these cocrystals, in a general way, increased the solubility of poorly soluble drugs, as well as increasing their stability. The RCM is a promising technique in the field of cocrystal engineering, and we can expect much research with optimal results in a near the future.

## Figures and Tables

**Table 1:** Examples of cocrystals obtained by the RCM.

Drug	Coformer	BCS	Improved Properties	Reference
Acyclovir	Fumaric acid	III	Solubility and permeability	Yan et al., 2013 [62]
Carbamazepine	Nicotinamide	II	Solubility	Rodríguez-Hornedo et al., 2006 [18]
Carbamazepine	17 Carboxylic Acids *	II	Solubility	Childs et al., 2008 [40]
Carbamazepine	Salicylic Acid, 4-Aminobenzoic Acid	II	Solubility	Bethune et al., 2009 [63]
Carbamazepine	Theophylline, Caffeine, Nicotinamide, Malonic Acid, Glutaric Acid, Saccharin, Oxalic Acid, Succinic Acid, Salicylic Acid	II	Solubility	Good and Rodríguez-Hornedo 2009 [45]
Carbamazepine	Succinic Acid	II	Solubility, Stability	Huang and Rodríguez-Hornedo 2011 [64]
Carbamazepine	Saccharin	II	Solubility	Alhalaweh et al., 2012 [61]
Carbamazepine	Saccharin, Salicylic Acid, 4- Aminobenzoic Acid monohydrate	II	Solubility	Lipert et al., 2016 [65]
Carbamazepine	Saccharin, Salicylic Acid	II	Solubility	Cao et al., 2018 [66]
Danazol	Hydroxybenxoic Acid, Vanillin	II	Solubility	Lipert et al., 2016 [65]
Ezetimibe	Methyl Paraben	II	Solubility	Sugandha et al., 2014 [67]
Gabapentin	3-Hydroxybenzoic Acid, 4-Hydroxybenzoic Acid, Salicylic Acid, 1-Hydroxy-2-Napthoic Acid, Mandelic Acid, Tartaric Acid, Malic Acid, (+)-Camphoric Acid, Gallic Acid	II	Solubility, Stability	Sreenivas Reddy et al., 2009 [68]
Gabapentin-lactam	4-Hydroxybenzoid Acid, 4-Aminobenzoic Acid, Benzoic Acid, Gentisic Acid, Fumaric Acid	II	Solubility	Maheshwari et al., 2012 [69]
Indomethacin	Saccharin	II	Solubility	Alhalaweh et al., 2012 [61]
Indomethacin	Saccharin	II	Solubility	Lipert et al., 2016 [65]
Isoniazid	Resveratrol	I/III	No advantage	Rosa et al., 2019 [70]
Ketoconazole	Adipic Acid, Fumaric Acid, Succinic Acid	II	Solubility	Chen and Rodríguez-Hornedo 2018 [71]
Lamotrigine	Nicotinamide	II	Solubility	Cavanagh et al., 2018 [72]
Meloxicam	Salicylic Acid and Maleic Acid	II	Solubility	Machado 2016 [73]
6-Mercaptopurine	Isonicotinamide	II	Dissolution and Bioavailability	Wang et al., 2015 [74]
Moxifloxacin	4-hydroxybenzoic Acid	I	Solubility and Dissolution	Martínez-Alejo et al., 2014 [75]
Ofloxacin	Tartaric Acid Derivatives	I	Efficient separation of racemic compounds	He et al., 2018 [76]
Paracetamol	Oxalic Acid, 4-Bipyridine Cocrystal	I	Tableting properties	Ahmed et al., 2017 [77]
Piroxicam	Saccharin	II	Solubility	Lipert et al., 2016 [65]
Posaconazole	4-Aminobenzoic Acid	II	Solubility	Kuminek et al., 2019 [78]
Salicylamide	Oxalic Acid	I	Dissolution	Surov et al., 2017 [79]
Tadalafil	Malonic Acid	II	Solubility	Shimpi et al., 2018 [80]
TAK-441	L-malic Acid, L-tartaric Acid	II	Solubility, Stability	Iwata et al., 2016 [81]
Theophylline	Nicotinamide, Salicylic Acid	I	Solubility	Good and Rodríguez-Hornedo 2009 [45]
Theophylline	Citric Acid	I	Solubility	Jayasankar et al., 2010 [82]

* Succinic Acid, Benzoic Acid, Ketoglutaric Acid, Maleic Acid, Glutaric Acid, Malonic Acid, Oxalic Acid, Camphoric Acid, 4-Hydroxybenzoic Acid, Salicylic Acid, 1-Hydroxy-2-naphthoic Acid, DL-Tartaric Acid, L-Tartaric Acid, Glycolic Acid, Fumaric Acid, DL-Malic Acid L-Malic Acid.
